# Effects of Sowing Date on Phenotypic Plasticity of Fitness-Related Traits in Two Annual Weeds on the Songnen Plain of China

**DOI:** 10.1371/journal.pone.0127795

**Published:** 2015-05-29

**Authors:** Haiyan Li, John L. Lindquist, Yunfei Yang

**Affiliations:** 1 Key Laboratory of Vegetation Ecology, Ministry of Education, Institute of Grassland Science, Northeast Normal University, Changchun, Jilin Province, P. R. China; 2 Department of Agronomy and Horticulture, University of Nebraska, Lincoln, Nebraska, United States of America; University of Thessaly, GREECE

## Abstract

**Background:**

Phenotypic plasticity of fitness-related traits is vital for plant species to adapt to variable environments. *Chenopodium glaucum* L. and *Amaranthus retroflexus* L. are two common weed species globally. Understanding the plasticity in life-history traits, especially in reproductive allocation, within and among these species is important for predicting their success and for managing them in different environments.

**Methodology/Principal Findings:**

Seeds of the two plant species were sown every 10 days from 26 Jun to 15 Aug. Life-history and fitness-related traits of both phenology and morphology were measured, and dry biomass of roots, stems, leaves, and reproductive tissues was determined at physiological maturity. Length of reproductive and total life period of the two species differed among six sowing-date treatments. Later germinating plants led to relatively reduced total life period, size, and earlier reproduction than earlier germinating plants. The ratio of reproductive biomass to total plant biomass increased with later planting dates in *C*. *glaucum* but declined in *A*. *retroflexus*. Mature plant height, crown diameter, and reproductive tissue biomass, and seed production of *C*. *glaucum* and *A*. *retroflexus* increased with delayed reproductive period. Both species displayed true plasticity in reproductive allocation. However, the sowing date had a far greater effect on rate of vegetative growth than on allocation to reproduction.

**Conclusions/Significance:**

The fitness of both *C*. *glaucum* and *A*. *retroflexus* populations have an apparent increase when the period between germination and seed production is much longer. However, *C*. *glaucum* appears better adapted to later sowing than *A*. *retroflexus*. Controlling seedlings prior to reproduction will alleviate the negative effect not only in the present year but also in future years.

## Introduction

Phenotypic plasticity and fitness-related traits are of vital significance for plant species to adapt to [1,2] or tolerate heterogeneous environments [3–5]. In the last two decades, phenotypic plasticity of plants has become a central issue of ecological and evolutionary research [6]. Early studies of plant phenotypic plasticity were limited to simple descriptions of morphological characteristics such as plant size and branch number [7,8]. Recent studies have focused on aspects of plasticity in life history characteristics such as allocation of biomass to various plant parts along natural or simulated environmental gradients [9–12] and the allometry of reproduction. Weiner et al. (2009) recently argued that much of the data considered as evidence for plasticity in reproductive allometry are actually evidence for plasticity in the rate of growth and development. True plasticity in allocation was defined as a change in the allometric relationship itself, rather than a change in the rate of growth. They argued that true plasticity can only be determined when individual plant reproductive biomass is related to vegetative biomass, as opposed to analyzing biomass ratios, because the former accounts for differences in the size among individuals resulting from multiple factors occurring during growth [13]. Further understanding of plasticity in life-history traits, especially on reproductive allocation, among species is important for predicting their success in different environments.

Annual plant species are commonly the most troublesome weeds in agricultural production and many early successional environments characterized by a high level of disturbance and environmental variability [11,14]. These weeds can frequently germinate early in the growing season and continue to germinate throughout the growing period, an adaptation that maximizes seed production and fitness across a broad range of environmental conditions [15]. Regardless of the length of time annual plants are able to grow, seed maturity often signals senescence and plant death. Plants that germinate later in the season tend to have a shorter overall life span and a particularly brief reproductive period. In nature, plants that germinate at different times also are faced with variable biotic and abiotic conditions. Therefore, in addition to life span and reproductive period, other plant life-history characteristics such as the timing of phenological events and biomass allocation to different plant components also may have important impacts on overall success in a particular environment [11,16].

*Chenopodium glaucum* L. and *Amaranthus retroflexus* L. are common weedy annuals in temperate regions throughout the world. In the northern regions of China, these two species are ubiquitous [17]. Both species grow rapidly, especially in the rainy, hot summer season and show high phenotypic plasticity in characteristics such as plant size and seed production in response to variable environments [18,19]. Their seedlings usually emerge over several months from early spring to early autumn in suitable habitats. Phenological development of *C*. *glaucum* and *A*. *retroflexus* have been shown to be photoperiod sensitive [20,21], suggesting that time of emergence may have strong impacts on their phenotypic plasticity. Zhou et al. (2005) studied the phenotypic plasticity of life history characteristics of these species in response to early-season emergence in late April, mid-June, or mid-July and found that delaying germination led to earlier onset of the reproductive stage of development and relatively greater reproductive effort (RE, the ratio of reproductive biomass to total plant biomass) [10]. In northeast China, rainfall occurs mainly from June through August, which would allow for later emergence and potential escape from weed management in cropping systems. However, it remains unclear how much phenotypic plasticity in fitness-related traits exists when these species emerge later, during the wettest period of the growing season. Plasticity of life history traits in response to time of germination is critical because it may influence the degree of genetic variation and the heritability that is expressed in those life history characteristics [12].

Owing to the importance of *C*. *glaucum* and *A*. *retroflexus* in disturbed habitats and their widespread distribution in temperate regions of the world, we selected them as model species for the study of phenotypic plasticity and reproduction. We expect that a deeper understanding of the plasticity of fitness-related characteristics will yield important insights into their ability to maintain population under various environmental conditions. Therefore, the primary objectives of this research were to determine the impact of late-season emergence date on the timing of phenological traits, biomass accumulation, and allocation of biomass in these species. We hypothesize that: (1) the reduction in total life period caused by delayed sowing will result in a greater proportion of the life cycle of both species being in the reproductive phase, (2) the reduced total life period of later sown plants will result in reduced height, crown diameter, and biomass of various organ groups, (3) reproductive effort will increase with delayed sowing, and (4) there is true plasticity in reproductive biomass allocation owing to changes in phenology in response to sowing date.

## Materials and Methods

### Species description

*Chenopodium glaucum* and *A*. *retroflexus* are annual dicotyledonous weeds. They are widely distributed in all temperate regions of the northern and southern hemispheres [17,22]. They are typically most successful in disturbed areas such as roadsides, croplands, wetlands, and even lawns. They grow rapidly, especially during the warm rainy season. Their stems are erect, 0.05–2 or even 3 m tall in favorable habitats. Inflorescences are usually densely grouped in panicles. They are short-day plants, and flowering generally occurs in early August with seeds ripening in September. A single plant can produce from several to several hundred thousand seeds. They are problem weeds for many crops and difficult to eradicate once established because of the abundant seeds in the soil seed bank [22,23].

### Study site

The study was conducted in 2009 at the Pasture Ecology Research Station of Northeast Normal University, Changling, in western Jilin province, China. The site, located at 44° 40′ N, 123° 44′ E, is in a flat (0–2% slopes) low-lying southern portion of the Songnen plain. Climate of the area is temperate, semi-arid continental monsoon with a mean annual temperature of 4.9°C and annual precipitation of 471 mm, the majority coming between June and August. Annual potential evapotranspiration is 1668 mm, 3.5 times the amount of precipitation. The growing season is comprised of approximately 150 frost free days between early May and late September.

### Experimental method

*Chenopodium glaucum* and *A*. *retroflexus* seeds were collected in the autumn of 2008 from wild populations growing at the research station, and stored dry at room temperature until the study was initiated. The experimental field was planted to corn in the previous year and some annual and biennial plants were present prior to initiation of the experiment. All vegetation was removed by hoe before planting. A randomized complete block experimental design was used with species and six sowing date treatments randomized within three replicate blocks. Each replicate block contains one 12 m^2^ (3 m × 4 m) experimental plot of each treatment type. To establish sowing date treatments, seeds of each species were sown on 26 June, 6 July, 16 July, 26 July, 5 August (the *C*. *glaucum* seeded in this treatment did not emerge, so they were replanted on 13 August), and 15 August. Seeds were sown by hand and gently raked to cover them with approximately 1 cm of soil. Plots were manually irrigated for several days after planting to ensure seedling emergence. Plots were thinned to the target density of one plant m^-2^ when plants uniformly reached the four-leaf stage. Our goal was to compare uniformly sized plants within a sowing date treatment among sowing dates. No fertilizer was added to the soil. *Chenopodium glaucum* and *A*. *retroflexus* plants were not obviously affected by insect pests throughout the study and all other plants were pulled by hand.

Over the course of the growing season, we recorded the date of initiation of key phenological events for each of ten uniformly sized plants per experimental plot. Phenological events included: (1) emergence, (2) initiation of inflorescences on the apical meristem (bolting date), (3) first observance of an open flower (flowering date), and (4) plant death. To assess the effects of planting date on the length of time each species spent within a phenological period, the average dates of these events within an experimental plot were used to calculate several time intervals (days): (1) emergence period (sowing to emergence), (2) vegetative period (emergence to bolting), (3) flowering interval (bolting to flowering date), (4) post-reproductive period (flowering date to death), (5) reproductive period (bolting to death), (6) life period (emergence to death), and (7) difference in total life period (difference between life period of any given treatment and life period of last sowing date treatment for each species (15 Aug)). The proportion of the total life period spent within each of these phenological time intervals was also calculated.

At the end of the growing season, when most *C*. *glaucum* and *A*. *retroflexus* seeds had matured but had not yet shattered (late September and early October), each of the ten plants per experimental plot were destructively and carefully harvested to obtain above- and below-ground biomass. Prior to harvest, height was measured from the soil surface to the apex of the plant and crown diameter was calculated as the average of the maximum lateral spread of the canopy and the lateral spread along an axis perpendicular to the maximum lateral spread. Soil was then excavated to a depth of 50 cm and the soil was washed free of roots. Each plant was then separated into roots, stems, leaves and reproductive tissues (including all parts of an inflorescence and seeds), bagged, and dried at 80°C to constant weight. Reproductive parts from all ten plants per experimental plot were then pooled, and a homogenized subsample of reproductive tissues from each experimental plot was weighed. From this, we separated and obtained the mass of 100 seeds. Seed production per plant was then estimated from the mass of reproductive tissues and the mass of 100 seeds within the subsample for each experimental plot (S1 and S2 Files).

### Data analysis

Preliminary data analysis compared the mean (M) time to occurence of the four phenological events, defined as when 50% of all plants in a plot reached that event. Mixed model one-way analysis of variance (ANOVA) was used to assess the differences of variables among planting date treatments within species (*P* < 0.05). Planting date treatment was considered a fixed effect and replicate block as the random effect. Differences among least squares treatment means for each variable were determined using Tukey’s multiple comparison test with a significance level of 0.05. Dependent variables were log transformed as needed to meet the assumptions of ANOVA. Dependent variables included phenological periods (vegetative period, flowering period, post-reproductive period, reproductive period and total life period), fitness-related traits (height, crown diameter, root, stem, leaf, reproductive tissue, and seed biomass, number of seeds produced and total biomass), proportion of life history period (vegetative period, flowering period, and post-reproductive period), and the ratio of of biomass within a group (root, stem, leaf, and reproductive tissue biomass) to total plant biomass. Overall mean height, crown diameter, and biomass of various organ groups were regressed on difference in total life period using linear, power or exponential functions.

Differences in the plasticity of reproductive allocation was quantified using analysis of covariance in SAS PROC GLM. Within a species and sowing date, reproductive effort is quantified as the slope of the relationship between log transformed per plant reproductive biomass and log transformed per plant vegetative biomass [13]. In doing so, we assume that reproductive biomass is primarily determined by vegetative biomass. Heterogeneity in slope value among sowing date treatments was assessed by including sowing date and the sowing date by log transformed vegetative biomass interaction term as covariates. A significant interaction term indicates heterogeneity of the (unequal) slope in the relationship between reproductive biomass and vegetative biomass [24], which means that the relative impact of vegetative biomass on reproductive biomass differs among sowing date treatments. Therefore, there is true plasticity in reproductive effort [13].

## Results

### Plasticity in phenology

**Fig 1:**
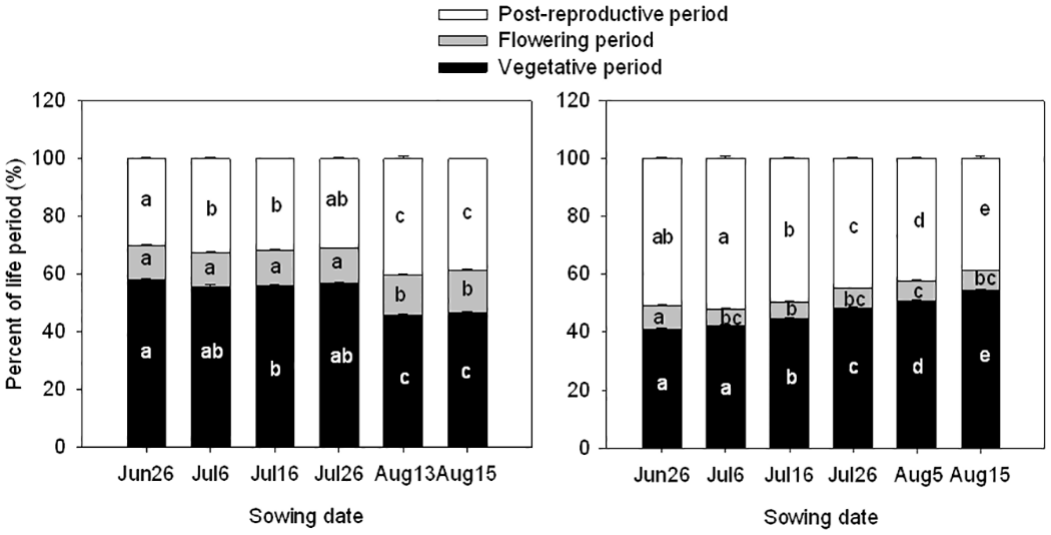
Differences in percent of life period occurring in the vegetative, flowering, and post-reproductive stages of development for *Chenopodium glaucum* (left panel) and *Amaranthus retroflexus* (right panel) as influenced by sowing date. Error bars are ±SE. Different letters for the same period of development indicate differences using Tukey’s multiple comparison test with a significance level of 0.05.

Sowing date had significant impacts on the timing of key phenological events, and more importantly on the period each species spent within a phenological growth stage (Table 1). *Chenopodium glaucum* and *A*. *retroflexus* seeds sown on June 26 and July 6 emerged 1 to 2 d later than later sown seeds (Table 1). Early sown *A*. *retroflexus* seeds emerged one day earlier (at 4 d after sowing) than *C*. *glaucum*. Vegetative period was substantially reduced with later sowing date and ranged from 52 to 24 d in *C*. *glaucum* and from 37 to 28 d in *A*. *retroflexus*. The flowering period, post-reproductive period, and reproductive periods also were reduced with later sowing in both species. *Chenopodium glaucum* had a longer vegetative period and shorter reproductive period than *A*. *retroflexus* from the June 26 to July 26 sowing date treatment. Total life period of *C*. *glaucum* and *A*. *retroflexus* was similar within a sowing date treatment, and declined in a similar manner with later sowing. Total life period of *C*. *glaucum* and *A*. *retroflexus* declined from 90 to 50 d and from 89 to 51 d, respectively. The percent of the total life period that *C*. *glaucum* spent in reproductive stages of development increased in the two latest sowing dates, whereas *A*. *retroflexus* showed a trend in the opposite direction (Fig 1). For both species, post-reproductive period was more affected than the flowering period. The difference in total life period among sowing date treatments was greater in the earliest sown treatments and decreased with later sowing dates for both species.

**Table 1:** Effect of sowing date on phenological traits and duration of development period (days) of *Chenopodium glaucum* and *Amaranthus retroflexus*.

Plant species	Sowing date	Emergence date	Bolting date	Flowering date	Death date	Emergence period (days)	Vegetative period (days)	Flowering period (days)	Post-reproductive period (days)	Reproductive period (days)	Life period (days)
*C*. *glaucum*	Jun 26	Jul 1	Aug 13	Aug 26	Sep 25	5	52.10 (0.63) a	10.77 (0.38) a	26.87 (0.45) a	37.63 (0.40) a	89.73 (0.41) a
	Jul 6	Jul 11	Aug 19	Aug 30	Sep 28	5	45.70 (0.68) b	9.63 (0.45) b	26.53 (0.39) a	36.17 (0.54) b	81.87 (0.36) b
	Jul 16	Jul 20	Aug 26	Sep 6	Sep 30	4	40.60 (0.19) c	9.13 (0.25) b	23.87 (0.18) b	33.00 (0.28) c	75.03 (0.27) c
	Jul 26	Jul 30	Sep 4	Sep 12	Oct 4	4	37.77 (0.35) d	8.07 (0.05) c	20.53 (0.37) c	28.60 (0.38) d	66.37 (0.11) d
	Aug 13	Aug 16	Sep 5	Sep 13	Oct 6	3	23.67 (0.38) e	7.43 (0.11) c	20.80 (0.38) c	28.23 (0.29) d	51.90 (0.16) e
	Aug 15	Aug 18	Sep 10	Sep 18	Oct 8	3	23.63 (0.10) e	7.40 (0.09) c	19.10 (0.12) c	26.50 (0.14) e	50.10 (0.18) f
*A*. *retroflexus*	Jun 26	Jun 30	Aug 1	Aug 10	Sep 25	4	36.50 (0.42) a	7.30 (0.29) a	45.07 (0.24) a	52.37 (0.32) a	88.87 (0.30) a
	Jul 6	Jul 11	Aug 9	Aug 13	Sep 27	4	33.83 (0.41) b	4.50 (0.39) b	41.87 (0.51) b	46.37 (0.30) b	80.20 (0.22) b
	Jul 16	Jul 20	Aug 17	Aug 22	Sep 29	4	33.13 (0.48) bc	4.40 (0.19) b	36.90 (0.20) c	41.30 (0.24) c	74.43 (0.33) c
	Jul 26	Jul 30	Aug 28	Sep 2	Oct 3	4	32.11 (0.28) c	4.35 (0.18) b	29.82 (0.19) d	34.19 (0.19) d	66.29 (0.20) d
	Aug 5	Aug 8	Sep 5	Sep 9	Oct 6	3	29.00 (0.27) d	4.03 (0.06) bc	24.14 (0.21) e	28.17 (0.22) e	57.17 (0.14) e
	Aug 15	Aug 18	Sep 13	Sep 16	Oct 8	3	27.97 (0.27) e	3.47 (0.10) c	19.90 (0.34) f	23.37 (0.27) f	51.33 (0.10) f

Emergence date, bolting date, flowering date, and death date indicate the date of initiation of each.

Emergence period, the interval from sowing date to emergence date (emergence date of each sowing was same, here all SE were zero). Vegetative period, the interval from emergence date to bolting date. Flowering period, the interval from bolting date to flowering date. Post-reproductive period, the interval from flowering date to death date. Reproductive period, the interval from bolting date to death date. Life period, the interval from emergence date to death date.

Values are mean with SE in parentheses.

Different letters for the same attribute for each plant species indicate significant difference among sowing dates at *P* < 0.05.

### Plasticity in plant growth and biomass allocation

**Fig 2:**
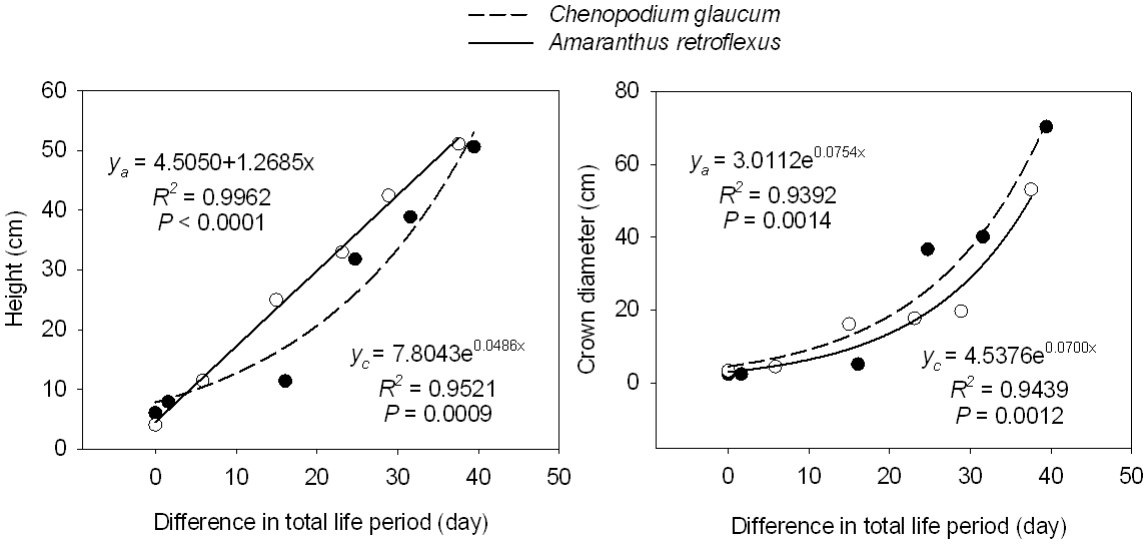
Height and crown diameter of *Chenopodium glaucum* (y_c_) and *Amaranthus retroflexus* (y_a_) in relation to the difference in total life period (difference between life period of any given treatment and life period of last sowing date treatment).

*Chenopodium glaucum* plant height declined from 51 cm in the June 26 sowing to 6 cm in the August 15 sowing, with the greatest decline occurring between the July 16 (32 cm) and July 26 (12 cm) sowing dates (Table 2). Similarly, *A*. *retroflexus* height declined from 51 cm to 4 cm in the first and last sowing dates, with the greatest decline occuring between the July 26 (25 cm) and August 5 (11 cm) sowing dates. Height of *C*. *glaucum* increased exponentially with difference in total life period, whereas *A*. *retroflexus* height showed a linear increase with increasing difference in total life period (Fig 2). *Chenopodium glaucum* average crown diameter declined from 70 to 2.3 cm in the first and last sowing dates, with the greatest decline between July 16 (37 cm) and July 26 (5 cm), and *A*. *retroflexus* diameter declined from 53 to 3.4 cm with the greatest decline between June 26 (53 cm) and July 6 (20 cm). Crown diameter of both species increased exponentially with increasing difference in total life period (Fig 2).

**Table 2:** Effect of sowing date on height, crown diameter, biomass accumulation, seed biomass, and seed production (seed number), total biomass, and the slope of the transformed reproductive biomass—vegetative biomass relationship (R-V) of *Chenopodium glaucum* and *Amaranthus retroflexus*.

		Sowing date					
Plant species	Attribute	Jun 26	Jul 6	Jul 16	Jul 26	Aug 13	Aug 15
*C*. *glaucum*	Height (cm)	50.60 (1.69) a	38.87 (0.88) b	31.80 (1.27) c	11.37 (0.40) d	7.88 (0.74) e	6.05 (0.43) e
	Crown diameter (cm)	70.36 (2.30) a	40.15 (1.85) b	36.66 (2.45) c	5.14 (0.77) d	2.41 (0.29) d	2.34 (0.13) d
	Root biomass (g)	3.559 (0.347) a	0.789 (0.125) b	0.656 (0.107) b	0.046 (0.006) c	0.017 (0.004) c	0.010 (0.001) c
	Stem biomass (g)	15.412 (1.824) a	2.996 (0.472) b	2.408 (0.387) b	0.087 (0.014) c	0.037 (0.008) c	0.017 (0.002) c
	Leaf biomass (g)	4.159 (0.381) a	1.164 (0.127) b	1.722 (0.242) c	0.130 (0.024) d	0.053 (0.011) d	0.033 (0.003) d
	Reproductive tissue biomass (g)	21.831 (2.351) a	5.526 (0.823) b	5.482 (0.897) b	0.387 (0.070) c	0.153 (0.027) c	0.088 (0.009) c
	Seed biomass (g)	14.399 (1.592) a	3.519 (0.571) b	3.335 (0.642) b	0.112 (0.019) c	0.048 (0.010) d	0.028 (0.005) d
	Seed number (seeds)	31234.3 (3454.2) a	22752.0 (3250.8) a	20094.4 (3128.6) a	203.1 (34.2) b	103.9 (22.8) bc	61.7 (10.4) c
	Total biomass (g)	44.962 (4.670) a	10.475 (1.497) b	10.268 (1.599) b	0.650 (0.108) c	0.260 (0.049) c	0.148 (0.015) c
	Slope R-V	1.06 (0.151) a	1.05 (0.137) a	0.97 (0.122) ab	0.78 (0.125) b	0.78 (0.121) b	0.87 (0.099) ab
*A*. *retroflexus*		Jun 26	Jul 6	Jul 16	Jul 26	Aug 5	Aug 15
	Height (cm)	51.10 (0.93) a	42.45 (1.06) b	32.98 (0.63) c	24.96 (0.76) d	11.43 (0.72) e	4.03 (0.23) f
	Crown diameter (cm)	53.12 (2.66) a	19.65 (2.35) b	17.71 (1.43) b	16.14 (1.28) b	4.48 (0.37) c	3.35 (0.18) c
	Root biomass (g)	1.470 (0.126) a	0.510 (0.046) b	0.340 (0.023) c	0.347 (0.027) c	0.091 (0.010) d	0.014 (0.002) d
	Stem biomass (g)	8.653 (0.793) a	3.072 (0.514) b	1.759 (0.153) c	1.343 (0.167) cd	0.227 (0.029) de	0.030 (0.003) e
	Leaf biomass (g)	5.576 (0.483) a	2.024 (0.240) b	2.338 (0.499) b	1.726 (0.197) b	0.361 (0.043) c	0.077 (0.008) c
	Reproductive tissue biomass (g)	20.011 (1.736) a	6.131 (0.636) b	5.482 (0.400) b	4.276 (0.413) b	0.619 (0.074) c	0.068 (0.007) c
	Seed biomass (g)	9.098 (0.805) a	2.510 (0.259) b	1.594 (0.153) bc	1.292 (0.173) c	0.092 (0.015) d	0
	Seed number (seeds)	27240.5 (2411.0) a	7503.7 (774.0) b	4406.4 (424.0) c	3546.6 (475.9) cd	284.9 (45.1) e	0
	Total biomass (g)	35.710 (3.064) a	11.737 (1.170) b	9.919 (0.837) b	7.692 (0.745) b	1.297 (0.144) c	0.189 (0.018) c
	Slope R-V	0.93 (0.175) ab	0.57 (0.156) c	0.57 (0.170) c	0.84 (0.165) ab	0.69 (0.153) bc	1.08 (0.122) a

Values are means with SE in parentheses. Different letters for the same attribute within a species indicate statistical difference among sowing date treatmetns at *P* < 0.05. Slope R-V is the slope of the regression of log transformed reproductive biomass on log transformed vegetative biomass. Intercept values were 0.139 and -0.312 and RMSE values were 0.111 and 0.117 for *C*. *glaucum* and *A*. *retroflexus*, respectively.

**Fig 3:**
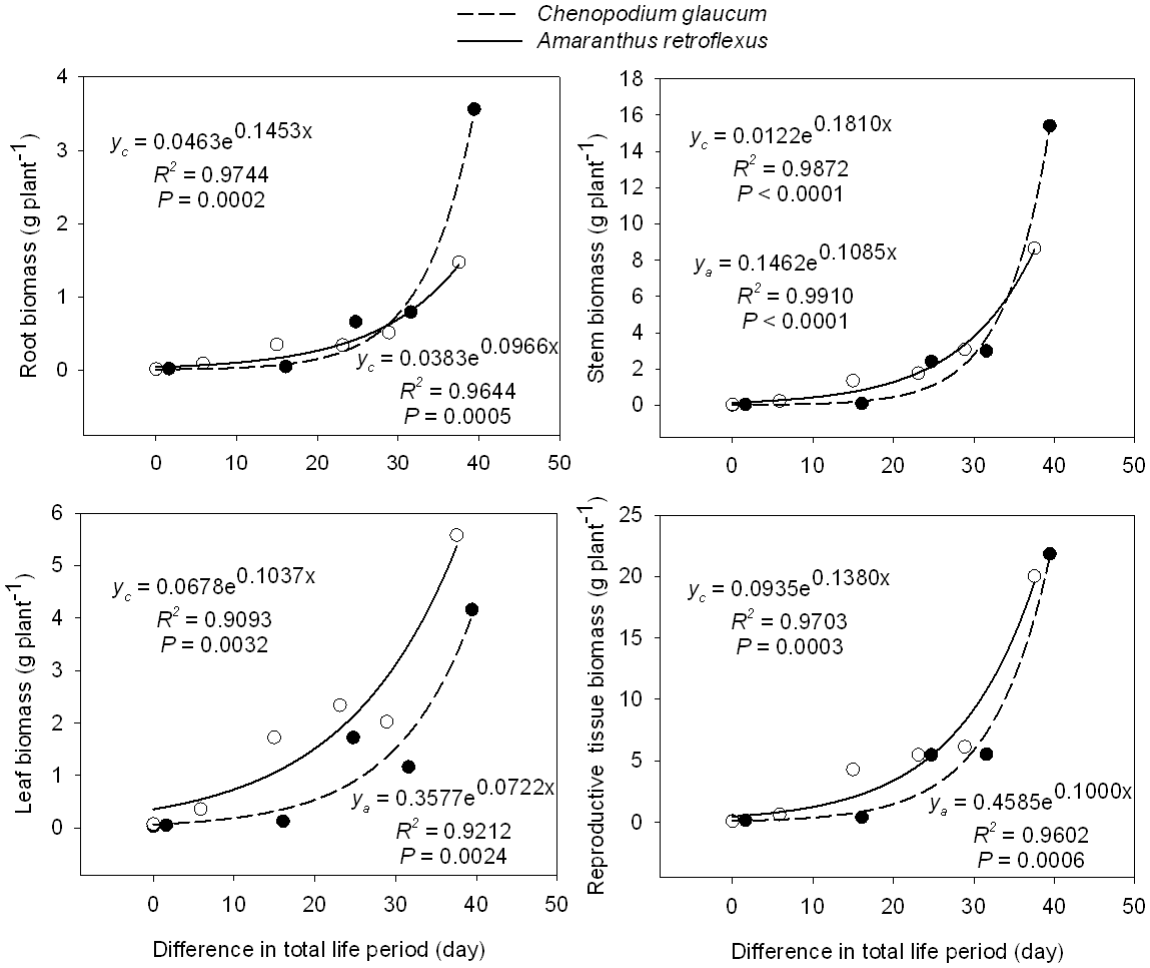
Biomass of various tissue groups of *Chenopodium glaucum* (y_c_) and *Amaranthus retroflexus* (y_a_) in relation to the difference in total life period (difference between life period of any given treatment and life period of last sowing date treatment).

**Fig 4:**
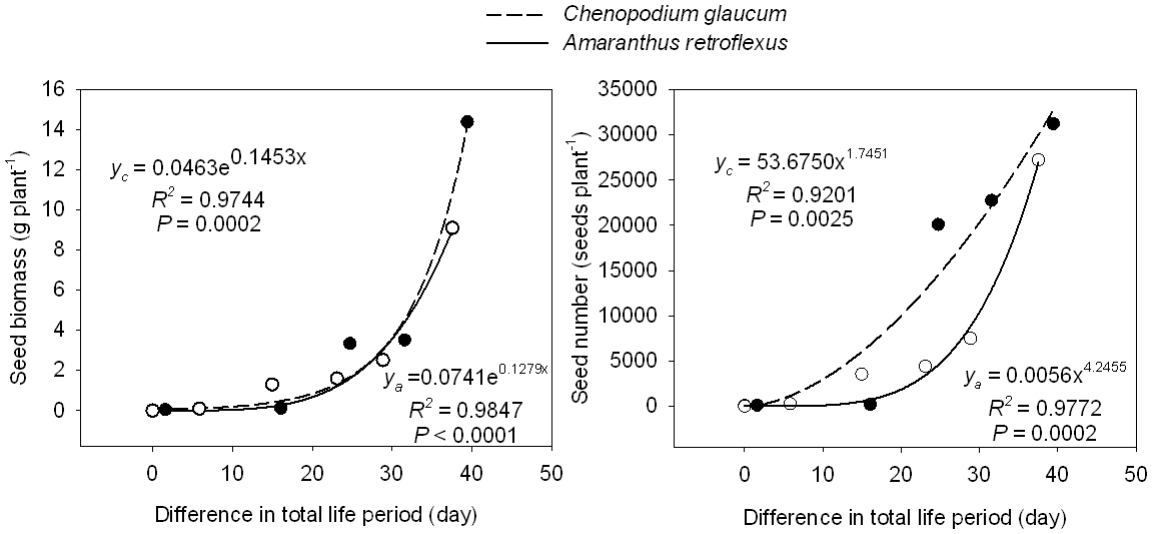
Biomass and number of seeds of *Chenopodium glaucum* (y_c_) and *Amaranthus retroflexus* (y_a_) in relation to the difference in total life period (difference between life period of any given treatment and life period of last sowing date treatment).

As with plant height and crown diameter, mature biomass of various plant components (root, stem, leaf, and reproductive tissue) declined with later sowing dates for both *C*. *glaucum* and *A*. *retroflexus* (Table 2). Maximum total plant biomass was 45 g plant^-1^ in *C*. *glaucum* and 36 g plant^-1^ in *A*. *retroflexus*. As with height and crown diameter, the greatest decline in biomass occurred between the June 26 and July 6 sowing dates in *C*. *glaucum* and *A*. *retroflexus*. With increasing difference in total life period of both species, biomass of roots, stems, leaves, reproductive tissues and seeds increased exponentially, but the number of seeds increased by a power function (Figs 3 and 4).

**Fig 5:**
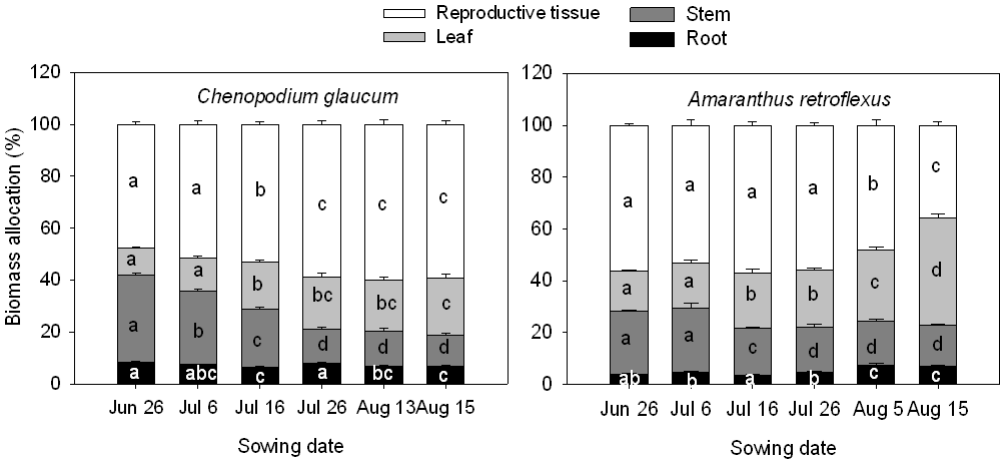
Results of multiple comparisons of differences in biomass allocation (% of total biomass, log transformed as needed) for *Chenopodium glaucum* and *Amaranthus retroflexus* among six sowing dates. **Error bars are ±SE.** Different letters for the same component indicate differences among sowing dates using Tukey’s multiple comparison test with a significance level of 0.05.

The proportion of total biomass in roots varied relatively little across sowing dates in both species (Fig 5). However, proportion of biomass in stem gradually declined and that in leaf and reproductive tissues increased with later sowing dates in *C*. *glaucum*. Proportion of total biomass of *A*. *retroflexus* stem also declined with later sowing dates, but the proportion in leaves increased so much at these later dates that the proportion of biomass in reproductive tissues actually declined with later sowing.

### Plasticity in reproductive effort

The mass of 100 *C*. *glaucum* seeds was 0.0483 g and did not vary among sowing date treatments. *Amaranthus retroflexus* seeds were smaller (0.0343 g per 100 seeds) and also did not vary among sowing date treatments. These seed masses compare favorably with those reported by Stevens [25]. Using the product of these masses and reproductive output, seed production per plant is shown in Table 2. While it was possible that some seeds may have been lost prior to plant harvest, care was taken to harvest plants prior to seed shed.

**Fig 6:**
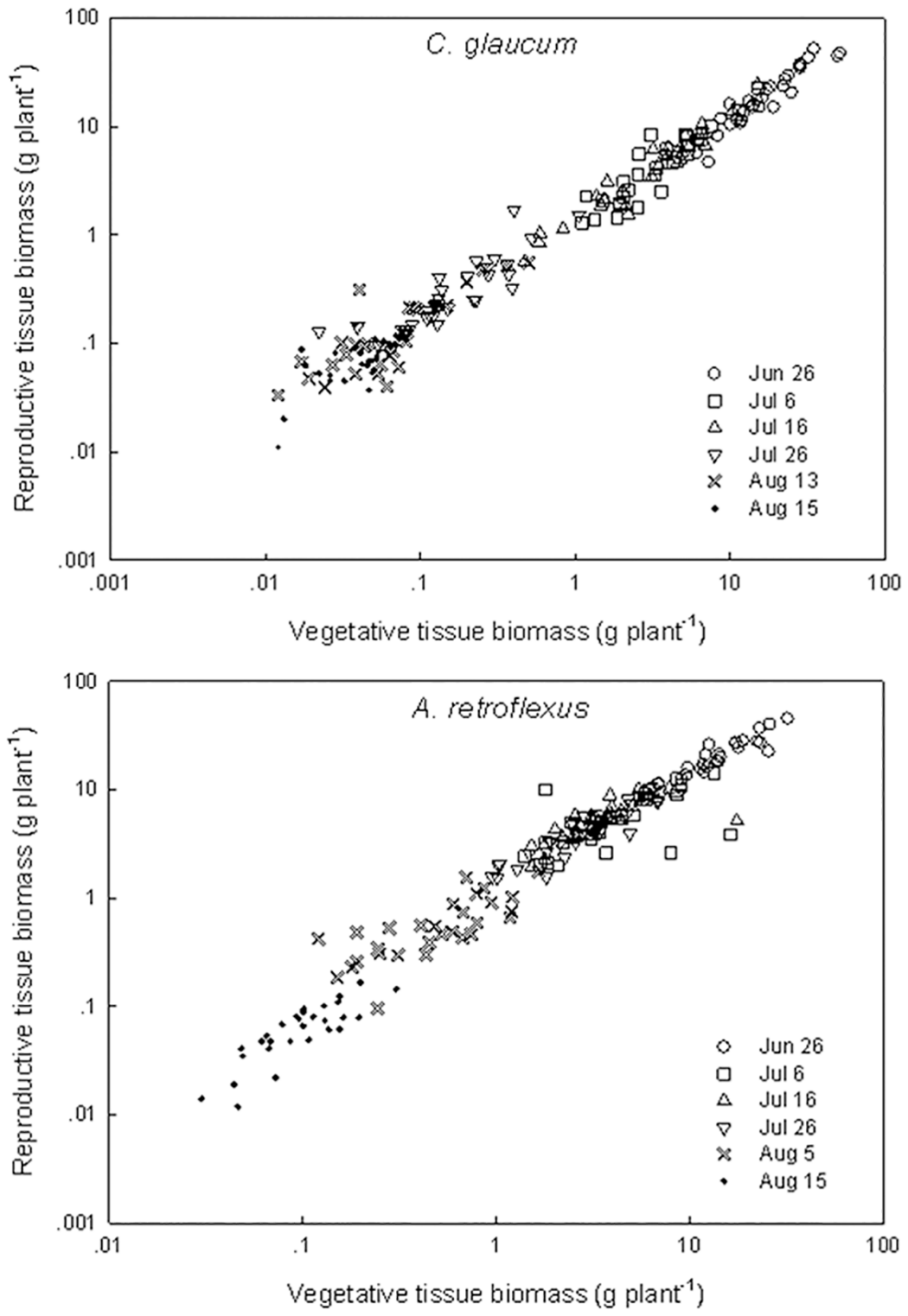
Relationship between biomass of reproductive tissue and total vegetative biomass on a log-log scale for *Chenopodium glaucum* and *Amaranthus retroflexus*.

Plasticity in allocation to reproduction is assessed by the relationship between reproductive biomass and total vegetative biomass (Fig 6), since this removes the effect of the duration of growth on total vegetative biomass [13]. For *C*. *glaucum*, the interaction effect of vegetative biomass and sowing date on reproductive biomass was significant (*F* = 2.25, *P* = 0.05), indicating differences in the slope of the relationship among sowing date treatments and true plasticity in reproductive effort (Table 2). The interaction effect of vegetative biomass and sowing date on reproductive biomass of *A*. *retroflexus* also was significant (*F* = 3.19, *P* = 0.009), indicating true plasticity in reproductive effort for this species.

## Discussion

### Plasticity in phenology within and among plant species

Plant life history characteristics vary widely within and among species, which allows species to thrive under varying environmental conditions [26]. In this study, sowing date treatments affected the amount of time that plants spent in each phenological stage in the life cycle of these species (Table 1). In later sown plants, reproductive tissues appeared earlier in their life span than in early sown plants (Fig 1). Plasticity in time of onset and period of reproduction has been documented in previous research [10,11,14,27]. Photoperiod has been reported as a primary environmental factor determining time of flowering in annual herbs, including *C*. *glaucum* and *A*. *retroflexus* [20,21,28–30]. We hypothesized that because of their photoperiod sensitivity, the reduction in total life period caused by delayed sowing will result in a smaller proportion of the life period in the vegetative stage and a greater proportion in the reproductive stages of development. This characteristic would allow for maximum fecundity even when the total life span is shorter. Total period of the life cycle ranged from about 90 d in the earliest sown treatments to about 50 d in the latest sown treatments for both species, which is as many as 30 days shorter than for these species when sown in late April [10]. Delayed sowing resulted in a reduced vegetative period relative to the total life period for *C*. *glaucum*, but not for *A*. *retroflexus* (Fig 1). Moreover, the proportion of the total life period spent in the reproductive stages increased with delayed sowing in *C*. *glaucum*, but not for *A*. *retroflexus*. A reduction in the proportion of total life span spent in reproduction for *A*. *retroflexus* is perplexing, because it could have a negative effect on seed production and, therefore, its long-term fitness. In a related study where *C*. *glaucum* and *A*. *retroflexus* were sown in early and late spring or early summer, Zhou et al. [10] found that later sown plants of both species increased the proportion of total life period spent in the reproductive stages. The shift toward less time spent in reproduction of *A*. *retroflexus* was only observed in plants sown after mid-July in our study. It appears that the response of *A*. *retroflexus* to the gradual decline in day length towards the end of growing season differs from that of *C*. *glaucum*. Therefore, *C*. *glaucum* may be better adapted to later emergence than *A*. *retroflexus*. Green foxtail (*Setaria viridis*), another widely distributed weedy annual in temperate regions also was shown to vary its life period with sowing date [11]. Similarly, soybean and *Puccinellia tenuiflora*, a perennial grass distributed on alkalized meadows in China, also expressed plasticity in life period with varying sowing dates [31,32]. This plasticity of life history allows plants to regulate the life period to successfully complete their life cycle and maintain population persistence even in unfavorable conditions.

### Plasticity in plant size

Sowing date also led to plasticity in morphological characteristics such as plant height and crown diameter (Table 2). We hypothesized that the reduced time spent in the vegetative phase of later sown plants would result in reduced height, crown diameter, and biomass of component plant parts. Indeed, later-sown plants harvested at the end of the growing season were shorter (only 4–6 cm) and crown diameter was considerably smaller (only 2.3–3.4 cm) (Fig 2) than early sown plants. Moreover, earlier sown plants produced greater total biomass, with much more biomass in roots, stem, leaves, and reproductive tissues resulting from the longer period of vegetative and reproductive growth (Fig 3). Biomass of component parts declined exponentially with later sowing date (i. e. greater difference in total life period). The reduced size of later sown plants also translated into smaller total seed biomass and number of seeds produced per plant (Fig 4). It is interesting that leaf biomass of both species was similar for plants in the earliest sowing date treatment, but as sowing date was delayed, *A*. *retroflexus* leaf biomass became increasingly greater than that of *C*. *glaucum*, suggesting that *A*. *retroflexus* was investing considerably more biomass in leaves than *C*. *glaucum*. This could partially explain the observation that *A*. *retroflexus* increased the proportion of total biomass in vegetative growth with later sowing compared to *C*. *Glaucum* (Fig 5). However, the reason for this is not clear. Huang et al. studied the effects of photoperiod on *C*. *album* [21] (a closely related species to *C*. *glaucum*) and *A*. *retroflexus* [20] in growth chambers. They grew these species in a 16 hr photoperiod for varying lengths of time, then transferred those plants to an 8 hr photoperiod and found that increasing the time to transfer from long to short day environments (i.e. longer periods of growth in long day environment) increased the number of leaves on plants, shoot height, shoot biomass, and the length of each stage of phenological development. They further found that when plants were grown in constant photoperiod, sensitivity to day length only differed when day length exceeded 14 or 12 hours for *C*. *album* or *A*. *retroflexus*, respectively. At the latitude of our research site, day length exceeded 12 hours every day from 23 March through 21 September and exceeded 14 hours between 2 May and 11 August. Longest day length (15.33 hours) occurred between 19 June and 24 June. If photoperiod is the sole driver of phenological development, then Huang et al’s [20,21] results suggest that both species should respond similarly to photoperiod in our environment. Therefore, it is possible that *A*. *retroflexus* was more sensitive than *C*. *glaucum* to the lower temperatures and reduced soil water availability late in the season at our research site.

Temperature and light quality also are strong drivers of phenological development [32]. Numerous studies have assessed the role of temperature or the accumulation of thermal units after plant emergence, but we know of no studies on the effects of late season temperature declines on phenological development. Since *A*. *retroflexus* is generally considered a warm-season C_4_ species, whereas *C*. *glaucum* is a cool season C_3_ species, it seems reasonable that the former would be more sensitive to cool temperatures late in the season. In a field study in the US, Knezevic et al. showed that the proportion of biomass partitioned to vegetative tissues during the reproductive phase of development increased, and partitioning to reproduction declined, with later *A*. *retroflexus* sowing date. However, they attributed the changes in biomass partitioning to shade avoidance resulting from interplant interference. Given the relatively wide spacing among plants in our study, the relative importance of interplant interference on *A*. *retroflexus* phenological development is not clear. Further research on the mechanisms controlling phenological development in these species when emerging late in the growing season is warranted.

### Plasticity in reproductive effort

Because the total life period was shorter with later sowing date, later sown plants were small and produced only a few leaves, but in general still managed to successfully produce a few seeds to maintain the population for another generation. These characteristics are typical for annual plants that are well adapted to varying environmental conditions. In favorable conditions, both vegetative and reproductive periods are maximized to maximize seed production and overall fitness [33]. In unfavorable environments, having a shorter life cycle, earlier flowering, and maximizing reproductive allocation provide a fitness advantage [10].

We hypothesized that the proportion of total biomass allocated to reproductive tissues will increase with delayed planting. Weiner et al. [13] indicate that the relationship between plant size and reproductive output is central to a plant’s strategy to convert growth into fitness. They argued that a plant with a given amount of resources at any point in time allocates those resources to different plant structures, and the allocation to reproductive effort has typically been quantified using reproductive effort (RE = reproductive biomass/total biomass). However, the RE ratio concept is assumed to be size independent when in fact reproductive allocation is very much size dependent. Moreover, plant size is determined by numerous interacting factors. Therefore, a calculated value of RE for plants within a given population at a specific time during growth may vary more as a result of differences in plant size than to true plasticity in reproductive allocation. A more accurate measure of reproductive allocation is the relationship between log transformed reproductive biomass and log transformed vegetative biomass because this takes into account whatever factors influenced plant size during growth. Therefore, while the biomass allocation to various organ groups as shown in Fig 5 is useful for assessing how much biomass is in those organ groups, it is not an accurate means of assessing true plasticity in reproduction. Owing to the wide range of total life period among sowing date treatments, we expected a large range in vegetative biomass across treatments in both of these species. We also hypothesized that there is true plasticity in biomass allocation owing to changes in phenology in response to sowing date. In particular, we expected that relatively more of the total biomass would be allocated to reproduction in the later sown smaller plants than larger plants. This phenomenon would be shown if the slope of the relationship between reproductive tissue biomass and vegetative tissue biomass (R-V) increased (i.e. more reproductive biomass per unit vegetative biomass produced) with later sowing dates. Fig 6 shows there was a great range in vegetative tissue biomass of individual plants of both species across sowing date treatments. At first look, it appears that reproductive tissue biomass is linearly related to vegetative biomass, regardless of sowing date treatment. However, slope of the relationship between reproductive tissue biomass and vegetative tissue biomass within a species also varied among sowing date treatment (Table 2), indicating true plasticity in biomass allocation across treatments. The largest slope value (1.06) for *C*. *glaucum* occurred in the earliest emerging treatment and slopes generally declined with later sowing date, which is opposite of what we expected. On the other hand, the largest slope (1.08) for *A*. *retroflexus* occurred in the latest emerging treatment, and the smallest slopes (0.57) occurred in intermediate sowing date treatments. Therefore, there is no clear trend in the changes in plasticity in biomass allocation across sowing date treatments for these two species. While the slope values of the R-V relationship differed significantly among sowing date treatments, it is not clear how biologically important those differences are relative to the effect of sowing date treatment on total plant biomass. In other words, the overall R-V relationship across all treatments appears to be far more important than the R-V relationship among individual treatments shown in Fig 6.

In *C*. *glaucum*, the proportion of its life cycle spent in the reproductive stages was longer and the proportion of total biomass in reproductive tissues increased with delayed sowing in our study, which is similar to the results shown by Zhou et al. [10]. However, the same was not true for *A*. *retroflexus* in our study, where a greater proportion of total biomass was in its leaves and less in reproductive tissues as sowing date increased. This result is contrary to the findings of Zhou et al. [10]. The reason for the apparent reduction in reproductive biomass in *A*. *retroflexus* may be a different response to late season temperature and soil water content compared to *C*. *glaucum*, though it is unclear why this would occur. It is clear that *A*. *retroflexus* allocated more biomass to leaves and less to reproduction with later emergence. As a result, some *A*. *retroflexus* plants didn’t produce mature seeds, which may have serious consequences for population growth under adverse circumstances.

The fitness of both *C*. *glaucum* and *A*. *retroflexus* populations increases when the reproductive and life periods are longer. Both species show plasticity in phenological development and biomass allocation in response to time of planting. Later germinating plants led to reduced total life period, plant biomass, and earlier reproduction than earlier germinating plants. Mature plant height, crown diameter, and reproductive tissue biomass of *C*. *glaucum* and *A*. *retroflexus* increased with increased difference in total life period (i.e. shorter life span). *Chenopodium glaucum* appears better adapted to later sowing than *A*. *retroflexus*.

### Effective weed control

*Chenopodium glaucum* and *A*. *retroflexus* are two pernicious weeds for many crops. They are difficult to eradicate once established because they produce abundant seeds and form a permanent seed bank in soil. The June 26 sowing in *C*. *glaucum* and *A*. *retroflexus* produced the greatest number of seeds with approximately 31,000 and 28,000 seeds per plant (Table 2), respectively, whereas the August 15 sowing resulted in the lowest seed production with approximately 60 and 3 unripe seeds per plant, respectively. Weed seed production provides valuable information for weed control [23]. The earlier the plants emerge, the more seeds are produced, and the more serious the potential adverse impact on crops in the following year. Our results suggest that controlling seedlings of these two plants prior to reproduction will reduce population growth and alleviate their negative effects on crop yield in future generations.

## Supporting Information

S1 File Phenological date, height, crown diameter and biomass data of *Chenopodium glaucum*. (XLS)

S2 File Phenological date, height, crown diameter and biomass data of *Amaranthus retroflexus*. (XLS)
